# Neural mechanisms underlying implicit emotion regulation deficit in relational and nonrelational trauma PTSD: Insights from the Nested Hierarchical Model of Self

**DOI:** 10.1017/S0033291725101505

**Published:** 2025-08-27

**Authors:** Yunxiao Guo, Qian Xiong, Yafei Tan, Junrong Zhao, Sijun Liu, Jiaojiao Jia, Zhihui Zhang, Yuyi Zhang, Zhihong Ren

**Affiliations:** 1 Key Laboratory of Adolescent Cyberpsychology and Behavior (Ministry of Education); 2 Key Laboratory of Human Development and Mental Health of Hubei Province, School of Psychology, Central China Normal University, Wuhan, China; 3 School of Psychology, Liaoning Normal University, Dalian 116029, China

**Keywords:** fusiform gyrus, implicit emotion regulation, posttraumatic stress disorder, relational trauma

## Abstract

**Background:**

Posttraumatic stress disorder (PTSD) exhibits marked heterogeneity, with relational (R; interpersonal) and nonrelational (NR; environmental) trauma subtypes demonstrating distinct psychopathological trajectories. Despite clinical recognition of these differences, their neurobiological underpinnings of emotion processing remain poorly understood. Guided by the Nested Hierarchical Model of Self (NHMS) – which posits trauma-type-specific disruptions in hierarchical self-processing systems – this study investigated neural mechanisms differentiating among PTSD subtypes during implicit emotion regulation.

**Methods:**

A sample of 122 participants, including patients with PTSD (R: *n* = 51; NR: *n* = 29) and trauma-exposed controls matched by trauma type (R: *n* = 22; NR: *n* = 20), underwent functional magnetic resonance imaging while performing the Shifted Attention Emotion Appraisal Task. Behavioral assessments and trauma typology coding were complemented by regions of interest (ROI)-based and whole-brain analyses.

**Results:**

Results revealed that PTSD-R showed hypoactivation in right superior frontal gyrus (during implicit emotion regulation; BA9; *p* = 0.049, ηp^2^ = 0.033), whereas PTSD-NR exhibited hyperactivation in fusiform (during emotion modulation by attention shifting; *p* = 0.036, ηp^2^ = 0.037). Symptom severity inversely correlated with social support (*r* = −0.353 to −0.417, *p* < 0.01), with relational PTSD reporting the lowest support (*p* < 0.001). Across conditions, dorsolateral prefrontal clusters (BA8/9) demonstrated anticorrelations with default-mode regions (*r* = −0.272 to −0.549, *p* < 0.01) aligning with NHMS’ predictive coding framework.

**Conclusions:**

These findings validate trauma-type-specific neural hierarchies, suggesting relational trauma disrupts top-down self-identity schemas, while NR trauma amplifies bottom-up threat detection. The study advances precision psychiatry by linking implicit regulation biomarkers to targeted interventions – cognitive restructuring for PTSD-R and interoceptive recalibration for PTSD-NR.

## Introduction

Posttraumatic stress disorder (PTSD) manifests through four core symptom clusters: intrusive recollections of traumatic events, persistent avoidance behaviors, marked alterations in cognitive-emotional processing, and sustained physiological hyperreactivity (Weathers et al., 2018). The updated International Classification of Diseases (ICD-11) (Zeigler-Hill & Shackelford, 2020) specifies that a diagnostic requirement for PTSD necessitates exposure to traumatic experiences defined as events or event sequences of profoundly threatening or horrifying nature (Barbano et al., 2019). The Diagnostic and Statistical Manual of Mental Disorders, Fifth Edition (DSM-5) explicitly proposes differential prognostic implications between relational and non-relational trauma subtypes (Forkus et al., 2023). Emerging research demonstrates that firsthand exposure to relational trauma (e.g. sexual abuse) coupled with childhood maltreatment significantly predicts the emergence of PTSD dissociative subtype, exhibiting a prevalence rate of approximately 14% in trauma-affected clinical populations (King et al., 2020). Although clinical evidence suggests that relational traumatic (PTSD-R) events (e.g. assault, torture, sexual violence) are associated with more severe and persistent symptom trajectories compared to nonrelational (PTSD-NR) catastrophic events (e.g. natural disasters, accidents), trauma causation lacks intentional deliberate human involvement (Scalabrini, Cavicchioli, Benedetti, Mucci, & Northoff, 2024).

Contemporary neuropsychodynamic frameworks systematically identify distinct psychopathological manifestations associated with different trauma categories, particularly in self-representation hierarchies (Mucci, 2021; Mucci & Scalabrini, 2021). Specifically, they propose that PTSD predominantly originates from agentive human harm (intentional violations of physical/psychological integrity) rather than accidental or natural traumatic events (Schimmenti & Caretti, 2016). It also implies that there are differences in the pathogenesis and manifestations of PTSD-NR versus PTSD-R traumatic experiences. Indeed, individuals are more likely to encounter a variety of trauma types, rather than a single event such as an earthquake or a major car accident (Contractor, Weiss, Natesan Batley, & Elhai, 2020; May & Wisco, 2016). The neuropsychodynamic frameworks are consistent with recent conceptualization of complex PTSD (CPTSD) as a distinct diagnostic entity (Maercker, 2021). The ICD-11 diagnostic criteria for CPTSD delineate self-organization impairments arising from prolonged exposure to inescapable relational traumas characterized by chronicity and multiplicity (Brewin et al., 2017).

Among the neuropsychodynamic frameworks, the Nested Hierarchical Model of Self (NHMS) provides a neurobiological framework to distinguish relational (e.g. abuse, betrayal) and nonrelational (e.g. accidents, disasters) trauma in PTSD (Scalabrini et al., 2024). A large-scale functional magnetic resonance imaging (fMRI) meta-analysis of healthy individuals identifies three hierarchical levels of self-processing: (1) interoceptive, which pertains to the processing of internal bodily states in response to external sensory stimuli; (2) extero-proprioceptive, which focuses on external and proprioceptive bodily inputs; and (3) mental, which addresses the internal cognitive processing of self-related versus nonself stimuli (Qin, Wang, & Northoff, 2020). Notably, the overlapping presence of interoceptive regions across all hierarchical levels demonstrates the self’s nested neural architecture, with core interoceptive areas being progressively integrated with domain-specific regions across processing levels. Based on this model, the NHMS further delineates three hierarchical self-processing tiers – interoceptive (anterior insula/dorsal anterior cingulate cortex [dACC]), extero-proprioceptive (temporoparietal junction [TPJ]/somatosensory cortices), and mental (medial prefrontal cortex [mPFC]/posterior cingulate cortex [PCC]) – to explain how trauma type modulates self-referential networks (Scalabrini et al., 2024). PTSD-R, marked by interpersonal violation, disproportionately disrupts the mental tier’s self-other demarcation, leading to altered mPFC activation during social evaluation and PCC-driven autobiographical memory intrusions (Lanius et al., 2017; Teicher, Samson, Anderson, & Ohashi, 2016). In contrast, PTSD-NR trauma often induces hyperactivation in the interoceptive tier (e.g. heightened insular response to bodily threats) and extero-proprioceptive dysfunction (e.g. TPJ-mediated spatial disorientation during flashbacks) (Hopper, Frewen, Van Der Kolk, & Lanius, 2007; Simmons et al., 2013). Critically, the model’s predictive coding architecture clarifies why relational trauma survivors exhibit top-down cognitive distortions (e.g. ‘I deserved abuse’), reflecting corrupted prior expectations in the mental tier, whereas nonrelational trauma frequently produces bottom-up interoceptive prediction errors (e.g. unexplained tachycardia in safe contexts) (Brewin, Gregory, Lipton, & Burgess, 2010). While this model suggests that interpersonal trauma tends to disrupt higher-order cognitive-affective integration (e.g. default mode network [DMN]), and nonrelational trauma may preferentially engage subcortical and interoceptive systems (e.g. insula, amygdala), this distinction is probabilistic rather than categorical. Relational trauma, especially in complex PTSD, often involves both top-down and bottom-up disruptions (e.g. self-schema distortions and hyperarousal). Recent evidence also supports that CPTSD is primarily rooted in chronic relational trauma, characterized by inescapable interpersonal harm and lasting self-organization impairments (Scalabrini et al., 2024). Thus, PTSD-R may involve overlapping dysregulation across multiple tiers of self-processing. This layered vulnerability underscores the value of a nested hierarchical rather than the binary model of trauma effects. This hierarchical differentiation informs targeted interventions: mental-tier remediation (e.g. cognitive restructuring) for relational trauma versus interoceptive recalibration (e.g. biofeedback) for nonrelational cases. By mapping trauma typology to specific neurocircuitry dysregulation, the NHMS advances precision psychiatry approaches for PTSD.

Our study aimed to provide further empirical evidence for the different neural mechanisms underlying emotion processing between relational and nonrelational trauma PTSD using an implicit emotion regulation task. Studies suggest that implicit emotion regulation deficits are a hallmark of PTSD (Kunimatsu, Yasaka, Akai, Kunimatsu, & Abe, 2020; Short, Boffa, Clancy, & Schmidt, 2018). Importantly, implicit emotion regulation should be distinguished from automatic emotional responses: the former refers to goal-directed but nonconscious modulation of affect, while the latter reflects stimulus-driven, nonregulatory processes (Braunstein, Gross, & Ochsner, 2017; Zhang, Bo, Wager, & Gross, 2025).

The Shifted Attention Emotion Appraisal Task (SEAT) (Duval et al., 2020; Zhao et al., 2025), used in this study, has proven to be a valuable tool for investigating implicit emotion in PTSD (Duval, Joshi, Russman Block, Abelson, & Liberzon, 2018; Liberzon et al., 2015).The SEAT task captures different levels of self-processing: attention-shifting targets lower tiers (e.g. interoceptive and extero-proprioceptive regions such as insula and TPJ), while appraisal taps mental-tier processes (e.g. mPFC, PCC), consistent with the NHMS framework (Qin et al., 2020; Scalabrini et al., 2024). By examining the neural differences in implicit emotion regulation between relational and nonrelational PTSD, researchers can identify biomarkers for PTSD subtypes, predict treatment outcomes, and develop interventions targeting the automatic emotional processes that underlie PTSD symptoms. The NHMS suggests that trauma typology differentially impacts hierarchically organized self-processing systems. To validate this framework, it is crucial to investigate the automatic neural mechanisms that function below conscious awareness – specifically, implicit emotion regulation. Implicit paradigms like SEAT (Duval et al., 2020) bypass volitional modulation, directly capturing how relational trauma corrupts mental-tier self-representations. From a social constructivist perspective, the development of the mental self – including self-reflection, perspective-taking, and narrative identity – is fundamentally shaped through early social interactions and relationships (Carpendale & Lewis, 2021). Relational trauma, which disrupts these formative interactions, is therefore more likely to impair higher-order self-functions such as autobiographical reasoning and social cognition. This level-specific engagement helps explain how PTSD-R disrupts mental-tier self-functions (e.g. narrative self and theory of mind), while PTSD-NR primarily affects sensory integration and interoceptive prediction systems (Barrett & Simmons, 2015; O’Brien, Felmingham, Lau, & O’Donnell, 2025). This approach aligns with NHMS’ predictive coding architecture: implicit tasks reveal trauma-induced mismatches between hierarchical expectations (stored in DMN) and automatic sensory inputs (processed in limbic regions) (Clark, 2018; Tivadar, Knight, & Tzovara, 2021). Together, our framework makes three key contributions: (1) distinguishing implicit regulation from automatic reactivity; (2) mapping SEAT conditions onto hierarchical self-processing tiers; and (3) linking PTSD subtype-specific neural disruptions to different layers of Implicit emotion regulation (O’Brien et al., 2025; Scalabrini et al., 2024). Thus, implicit regulation paradigms are not merely complementary – they are essential for elucidating NHMS’ hierarchical trauma typology.

Building upon NHMS (Qin et al., 2020), this study tests a hierarchical framework where trauma typology determines the neural level of dysregulation: PTSD-R trauma disrupts higher-order self-schemas through DMN alterations, while PTSD-NR trauma predominantly affects survival circuits via limbic hyperactivity. We hypothesize that trauma type differentially modulates neural systems involved in implicit emotion regulation: PTSD-R will show greater disruption in higher-order self-representational circuits (including anterior and posterior DMN regions such as the mPFC and PCC), while PTSD-NR will exhibit increased activation in lower-level sensory-survival circuits (e.g. insula, amygdala). This distinction reflects differential engagement of top-down versus bottom-up regulatory pathways during implicit emotional processing. Although prior research suggests that interpersonal trauma may confer elevated risk for PTSD development compared to noninterpersonal trauma (e.g. Brewin et al., 2017), the present cross-sectional design does not permit direct causal inference. Therefore, we present this consideration only as background rationale for our trauma type stratification, rather than as a testable hypothesis in the current study.

## Materials and methods

### Participants

**Figure 1.**
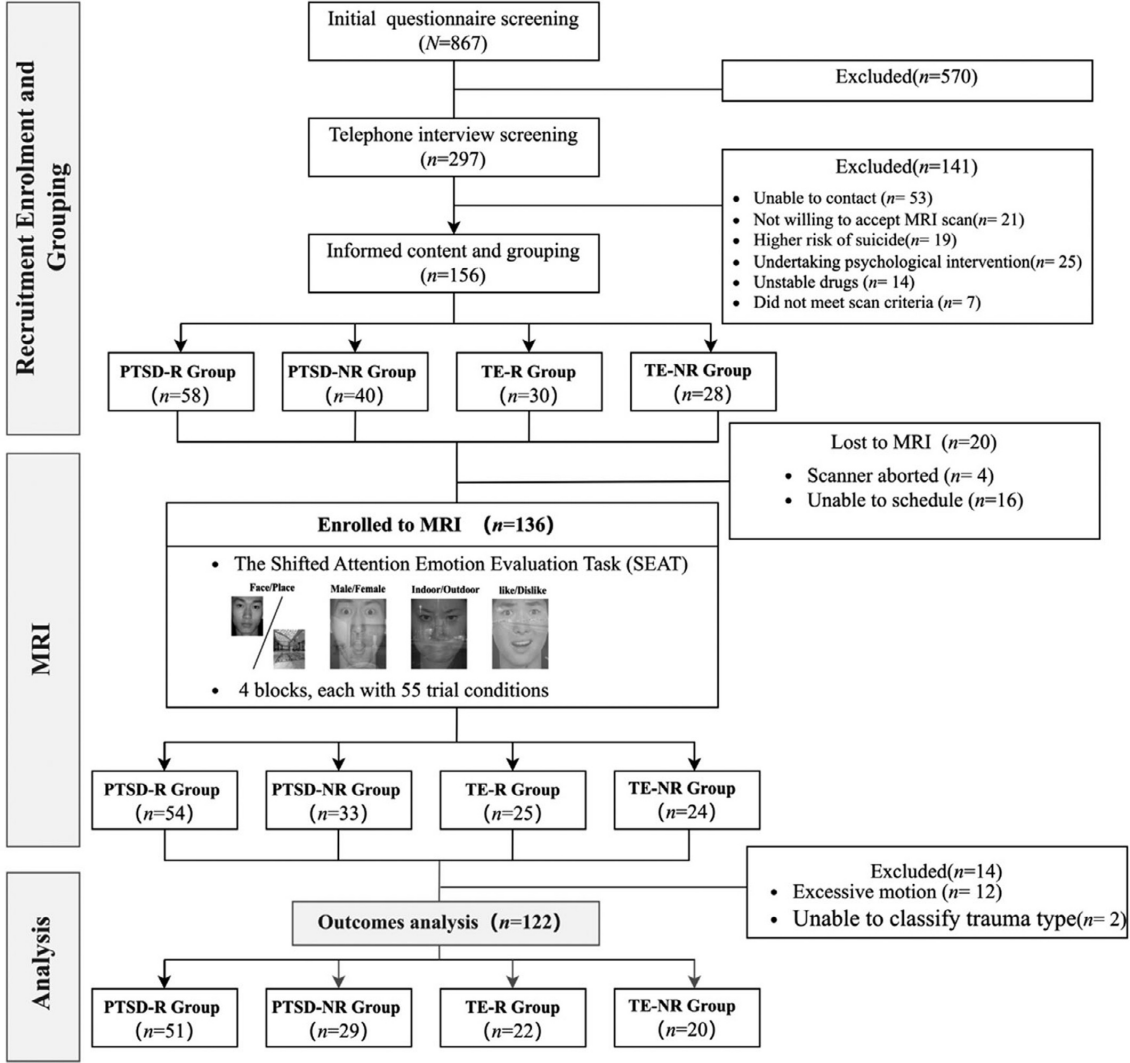
Research flowchart.

Of 297 interviewed participants, 122 were included in the final fMRI analysis (PTSD: *n* = 80; trauma-exposed controls: *n* = 42) after applying inclusion/exclusion criteria and accounting for exclusions due to scheduling, withdrawal, motion artifacts, and unclear trauma events. A flowchart of participant selection is shown in Figure 1. Participants were initially screened using the PTSD Checklist for DSM-5 (PCL-5) (cutoff ≥31), but final diagnostic grouping was based on DSM-5 criteria, confirmed through structured clinical interviews. These interviews were conducted by psychiatrists at Wuhan Psychological Hospital, with assistance from trained clinical and counseling psychology graduate students. Consensus diagnoses were established regarding PTSD status, psychiatric comorbidities (e.g. depression, anxiety), insomnia symptoms, substance use, crisis risk (e.g. suicidal ideation), treatment history, clinical stability, and fMRI eligibility. This assessment process ensured the exclusion of individuals with severe cognitive impairment, acute psychiatric instability, or ongoing psychotherapeutic intervention. Participants meeting full DSM-5 diagnostic criteria for PTSD were placed in the PTSD group, while those exposed to criterion A trauma but not meeting diagnostic threshold were categorized as trauma-exposed controls. Detailed inclusion/exclusion criteria and selection steps are provided in Supplementary Material A7. The study was approved by the ethics institutional review board of Central China Normal University and registered with the Chinese Clinical Trial Registry (Zhao, Zhao, Ren, Shi, & Lai, 2022).

### Totality measures

At the outset, prior to the interviews, we collected self-reported demographic information (gender, age, region, education) and administered the Posttraumatic Stress Disorder Checklist for DSM-5 (PCL-5), Generalized Anxiety Disorder 7-Item Scale (GAD-7), Patient Health Questionnaire-9 (PHQ-9), and The Medical Outcomes Social Support Scale (MOS-SSS).

### Clinical measurement

- PTSD: PTSD severity was assessed using the PCL-5 (Blevins et al., 2015), which demonstrates high internal consistency (Cronbach’s α = 0.94), excellent discriminant validity, and sensitivity to clinical change (Weathers, 2017). Participants with scores above 31 were included. The PCL-5 measures the four core PTSD symptoms: intrusion, avoidance, negative alterations in mood and cognition, and hyperarousal, with higher scores indicating greater symptom severity.
- Anxiety: Anxiety was evaluated using GAD-7 scale (Cronbach’s α = 0.92) (Hinz et al., 2017), where higher scores indicate greater anxiety.
- Depression: Depression symptoms were assessed using PHQ-9 (Kroenke, Spitzer, & Williams, 2001), with Cronbach’s α = 0.89 (Kroenke & Spitzer, 2002), where higher scores indicate more severe symptoms.
- Social support: MOS-SSS is a 19-item, self-administered social support survey developed for patients in the MOS (Sherbourne & Stewart, 1991), with Cronbach’s α = 0.96. This instrument was selected based on the stress-buffering hypothesis (Cohen & Wills, n.d.), which posits that perceived support mitigates trauma-related psychological distress. Recent meta-analyses also highlight that perceived – rather than structural – support is more strongly associated with PTSD symptoms, especially in interpersonal trauma contexts (Wang, Chung, Wang, Yu, & Kenardy, 2021). The higher the score, the more types of social support an individual can obtain and the higher the frequency.
- Classification of trauma types: Trauma typology was classified through a structured, multistep protocol grounded in DSM-5 criteria and trauma taxonomy frameworks (Alisic et al., 2014; Kerig, Ward, Vanderzee, & Arnzen Moeddel, 2009; Vibhakar, Allen, Gee, & Meiser-Stedman, 2019). To enhance ecological validity, item 17 of the Life Events Checklist for DSM-5 (LEC-5; Contractor et al., 2020) was modified to explicitly capture pandemic-related stressors (e.g. COVID-19 outbreaks), reflecting Wuhan’s unique epidemiological context. Participants’ self-reported trauma exposure was evaluated using: (1) LEC-5: standardized screening of 17 criterion A events. (2) Life Events Checklist Questionnaire for DSM-5 (LEC-5) (LECQT): open-ended questions to detect non-criterion A events with persistent distress (e.g. intrusive memories, avoidance). Events were prioritized hierarchically: self-experienced (Criterion A1) > witnessed (A2) > vicariously learned (A3). When multiple traumas were endorsed, we applied a predominance rule (>50% of events in one category) to assign trauma type. Ambiguous cases were resolved through consensus review. Traumas were coded into two categories based on World Health Organization (WHO) violence taxonomy and recent interpersonal trauma models (Hughesdon et al., 2021; Krug et al., 2000): (a) Interpersonal trauma (relational): events involving intentional human perpetration (e.g. abuse, sexual violence, betrayal) and (b) noninterpersonal trauma (nonrelational): events stemming from accidental or environmental causes (e.g. disasters, medical trauma).

Three PTSD-specialized clinicians independently coded events as interpersonal (intentional human perpetration: abuse, violence, betrayal trauma) or noninterpersonal (environmental/accidental: disasters, medical trauma), following WHO’s violence taxonomy relational dysfunction framework. Data were independently coded by three raters with both clinical and academic graduate students in PTSD. Any disagreements (<10% of cases) in ratings were discussed until a consensus was reached. Inter-rater reliability (prior to consensus discussions) was excellent, with a kappa of 0.846 (Lilleyman, 1992). Final groupings (PTSD-R, PTSD-NR, trauma-exposed-R, trauma-exposed-NR) were used for mixed analysis of variance (ANOVA) comparisons across clinical and neural outcomes. This procedure ensures diagnostic precision and reproducibility. Detailed classification criteria and trauma data are in Supplementary Material A1.2.3.

### MRI data acquisition

The study was conducted at Wuhan Psychological Hospital (April 2023–April 2024), where four participant groups underwent MRI scans while completing the SEAT, designed to probe neural mechanisms of implicit emotion regulation, attention shifting, and appraisal-based modulation (see Supplementary Material A2 for task details). Participants viewed composite images of emotional faces (angry, fearful, neutral) superimposed on indoor or outdoor scenes and responded to one of three cues presented before each image: ‘male/female’ (identify the face’s gender), probing implicit emotional processing; ‘indoor/outdoor’ (identify the scene location), probing emotion modulation by attention shifting; and ‘like/dislike’ (evaluate facial preference), probing emotion modulation by appraisal. Each of the 60 composite images was presented three times (once per condition), totaling 180 composite trials. Additionally, 40 noncomposite images (neutral faces or scenes alone) were presented to control for low-level visual processing, for a total of 220 trials across four randomized blocks. On each trial, participants first viewed a fixation cross (3–8 s), followed by a 750-ms cue, a 250-ms blank screen, and then the composite image for 1500 ms. Responses required either classification (e.g. gender or location) or evaluation (like/dislike), depending on the cue. Flowchart of the SEAT experimental paradigm (see Figure 1 in Supplemental Material A2). A trained MRI technician provided instructions, and safety checks were conducted. Participants remained still and awake during scanning. Data were collected using a Philips 3.0 T magnetic resonance scanner at Wuhan Psychological Hospital, China. Functional images were acquired using a gradient echo planar imaging sequence with the following scanning parameters: repetition time (TR)/echo time (TE) = 2000/30 ms, matrix = 64 × 64, FOV = 240 mm × 240 mm, flip angle = 90°, slice thickness/spacing = 4/1 mm. A total of 100 time points were collected. For the functional images, the slice thickness was 3.5 mm, with dynamic scan parameters of 240 scans and a voxel size of 3.5 mm × 3.5 mm × 3.5 mm.

### Statistical analysis

#### Participant characteristics and clinical symptoms analysis

Continuous variables were analyzed using one-way ANOVA with LSD post-hoc tests when meeting homogeneity of variance (assessed by Levene’s test), otherwise, Kruskal–Wallis H tests with Dunn–Bonferroni correction were applied. Categorical variables were compared using Pearson χ^2^ tests or Fisher’s exact tests where appropriate.

#### Data preprocessing

The results presented in this article were derived from preprocessing performed using fMRIPrep 23.1.4 (Esteban et al., 2019; RRID: SCR_016216), based on Nipype 1.8.6 (Gorgolewski et al., 2011; RRID: SCR_002502). Detailed preprocessing procedures are provided in Supplementary Material A6. MRI data analysis was conducted using Statistical Parametric Mapping (SPM12; Wellcome Centre for Human Neuroimaging, London, UK) in MATLAB. Runs with motion exceeding 3 mm in any direction (*x*, *y*, *z*, pitch, roll, yaw) were excluded from further analysis. Data were spatially smoothed with an 8-mm kernel. All motion parameters and their derivatives were included as nuisance regressors in the subject-level analysis.

#### Whole-brain data analysis

Eigenvariates from peak voxels were extracted using SPM and analyzed via 2 × 2 mixed ANOVA. Pearson’s correlations examined psychometric associations, with significance set at *p* < 0.05 and moderate-to-large effect sizes (|*r*| > 0.24). To isolate brain circuits associated with different emotion regulation processes, In the first-level analysis, we created three specific contrasts for each participant, in line with prior studies using the SEAT (Duval et al., 2018; Liberzon et al., 2015; Wang et al., 2016; Zhao et al., 2025): (1) male/female versus only neutral faces; (2) indoor/outdoor versus male/female; and (3) like/dislike versus male/female. Next, MRI data analysis was performed using Statistical Parametric Mapping (SPM12; Wellcome Centre for Human Neuroimaging) in MATLAB. Contrast images from the first-level comparisons were subsequently carried forward to second-level analyses. A one-sample t-test was conducted on the whole-brain data from all participants (*N* = 124) to identify the brain regions successfully activated by the SEAT paradigm. For all analyses, gender was included as a covariate of no interest. Statistical thresholds were set using Gaussian random field theory correction, with a voxel threshold of *p* < 0.005 and a cluster threshold of *p* < 0.05, following the recommendations of Eklund, Nichols, and Knutsson (2016) and Yan, Wang, Zuo, and Zang (2016)). Activated brain regions were reported using Brodmann areas and ALL3 templates via DPABI in MATLAB.

#### ROI analysis

To confirm that the SEAT task robustly engaged implicit emotion regulation regions, we first conducted a one-sample t-test across all participants (PTSD and trauma-exposed controls combined) to identify task-responsive activation clusters. This ensured functional validity of subsequent ROI analyses, as the SEAT involves complex multirun designs and a large number of trials (four runs, 220 trials total), following previous studies using this paradigm (Duval et al., 2020; Klumpp, Angstadt, & Phan, 2012; Sripada et al., 2012; Zhao et al., 2025). Eleven peak activation clusters were identified and defined as 6-mm radius spheres centered at group-level maxima. Beta values from each ROI were extracted using DPABI in MATLAB for each participant and each condition. These values were then analyzed using 2 × 2 mixed ANOVAs (trauma type × PTSD diagnosis) to examine group effects, consistent with our hypotheses on trauma-specific neural mechanisms. This two-step ROI-based strategy allowed us to maximize statistical power while retaining sensitivity to group-level differences in activation patterns.

## Results

### Participant characteristics

The study included 122 participants categorized into four groups: PTSD-R (*n* = 51), PTSD-NR (*n* = 29), TE-R (*n* = 22), and TE-NR (*n* = 20). Demographic characteristics and group comparisons are presented in Table 1. Significant between-group differences emerged in age (H = 58.540, *p* < 0.001), PTSD symptoms (PCL-5: *F* = 175.243, *p* < 0.001), anxiety (GAD-7: *F* = 47.856, *p* < 0.001), depression (PHQ-9: *F* = 61.176, *p* < 0.001), and social support (*F* = 8.503, *p* < 0.001). Post-hoc analyses revealed expected patterns: PTSD groups showed elevated psychopathology scores compared to trauma-exposed controls (all *p* < 0.001). No significant differences were found in gender distribution (χ^2^ = 2.544, *p* = 0.467), geographic region (Fisher’s exact *p* = 0.482), or education level (Fisher’s exact *p* = 0.163). Distributions of PTSD-R versus PTSD-NR trauma types are illustrated in Figure 3.

**Table 1.** Demographics and symptoms of PTSD-R group, PTSD-NR group, TE-NR group, and the TE-R group

Variables	PTSD-R group (*n* = 51)		PTSD-NR group (*n* = 29)		TE-R group (*n* = 22)		TE-NR group (*n* = 20)		Analysis of variance		
	*M*	*SD*	*M*	*SD*	*M*	*SD*	*M*	*SD*	χ^2^/H	*df*	*p*_value_
Age	22.12	4.403	20.86	2.310	42.00	13.306	35.20	11.821	58.540	3,118	<0.001^***^
Gender									2.544	3	0.467
Male	17	33.33%	14	48.28%	9	40.91%	10	50.00%			
Fame	34	66.67%	15	51.72%	13	59.09%	10	50.00%			
Region									5.127		0.482
Urban	38	74.51%	26	89.66%	15	68.18%	17	85.00%			
Suburban	3	5.88%	0	0.00%	1	4.55%	0	0.00%			
Rural	10	19.61%	3	10.34%	6	27.27%	3	15.00%			
Education									11.150		0.163
High school/vocational school and below	2	3.92%	1	3.45%	0	0.00%	1	5.00%			
Associate degree	3	5.88%	2	6.90%	1	4.55%	1	5.00%			
Bachelor’s degree	42	82.35%	23	79.31%	21	95.45%	12	60.00%			
Master’s degree and above	4	7.84%	3	10.34%	0	0.00%	6	30.00%			
PCL–5	43.10	10.167	42.66	9.656	6.05	3.592	5.65	4.870	82.148	3,118	<0.001^***^
GAD–7	11.02	4.785	10.83	4.986	2.00	1.309	1.45	1.572	76.620	3,118	<0.001^***^
PHQ–9	13.86	4.891	12.97	5.791	2.45	1.471	2.10	1.944	81.455	3,118	<0.001^***^
Social support	48.35	14.172	52.10	13.618	68.36	18.201	57.30	20.210	8.503	3,118	<0.001^***^

*Note:* Significance values are based on two-tailed tests. χ^2^, the result of chi-square test or Fisher–Freeman–Halton; GAD-7, Generalized Anxiety Disorder 7-item; H, the result of Kruskal–Wallis ANOVA; PCL-5, PTSD Checklist for DSM-5; PHQ-9, Patient Health Questionnaire-9;TE-NR, trauma exposure-non-relational trauma; TE-R, trauma exposure-relational trauma.

*** *p* < 0.001.

### Participant clinical symptoms analysis

Patients with PTSD exhibited significantly higher symptom severity (PCL-5), anxiety (GAD-7), and depression (PHQ-9) than trauma-exposed controls, regardless of trauma type. Social support was significantly lower in the PTSD group, with relational trauma cases showing the greatest disparity. No significant trauma-type effects emerged for symptom severity, anxiety, or depression. Detailed results are available in Supplementary Material A5.

### Activation of SEAT (whole-brain results)

**Figure 2.**
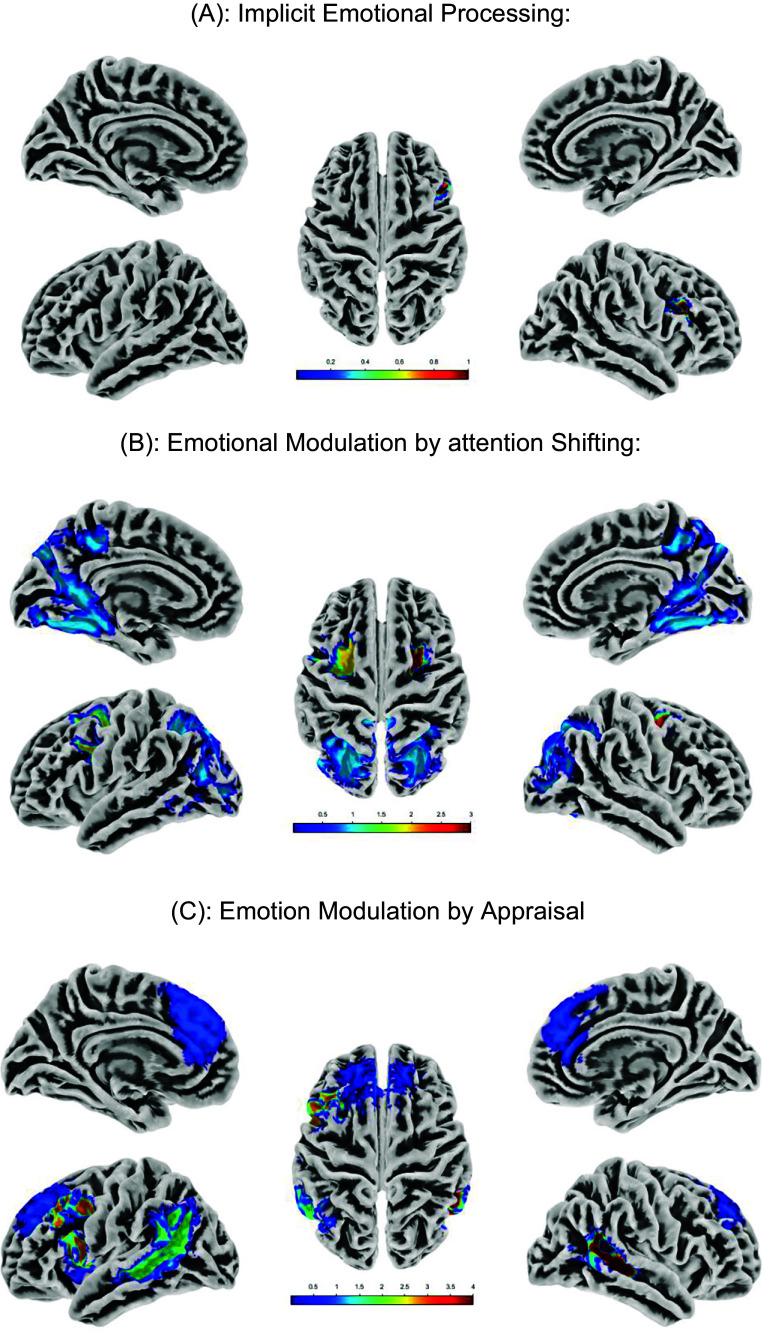
Group-level activation maps for SEAT task contrasts across all participants (*N* = 122), derived from one-sample t-tests. (A) Implicit emotional processing. (B): Emotional modulation by attention shifting. (C) Emotion modulation by appraisal.

During implicit emotion processing (male/female > face/place), significant activations were observed in regions associated, such as the dorsolateral prefrontal cortex (dlPFC), alongside deactivations in areas related to emotion regulation, including the dlPFC and fusiform gyrus. During emotion modulation through attention shifting (indoor/outdoor > male/female contrast), predicted activations were observed in regions involved in attention regulation (bilateral dlPFC), and deactivations were seen in areas associated with emotion processing (bilateral vmPFC; Figure 2B). During emotion modulation through appraisal (like/dislike > male/female contrast), predicted activations were found in both emotion processing and regulation regions (left dlPFC, Frontal_Mid; Figure 2C). Brain regions identified by one-sample t-tests across all participants (N = 122) were used for defining regions of interest (ROIs; Table 2).

**Table 2.** Brain regions identified by one-sample t-test across all participants (*N* = 122), used for defining regions of interest (ROIs)

		MNI coordinates					
Contrast	Anatomical area	*x*	*y*	*z*	*K*_E_	*t*	*p*_FWE_
Implicit emotional processing	ROI1: ACC (L)	−26.5	−45	−12	7687	−9.38	<0.001
	ROI2: insula (R)	43.5	21.5	23	121	6.13	0.013
	ROI3: dlPFC (R): Right Frontal_Sup_2	26	14.5	58	280	−5.97	<0.001
Emotional modulation by attention shifting	ROI4: left fusiform	−30	−38	−15.5	4030	12.80	<0.001
	ROI5: dlPFC (L)	−26.5	11	51	457	6.92	<0.001
	ROI6: mPFC (L) Frontal_Sup_Media	−9	53	30	438	−9.02	<0.001
	ROI7: dlPFC (R)	26	11	54.5	154	6.30	0.006
Emotion modulation by appraisal	ROI8: mPFC (L) Frontal_Sup_Media	−9	53	30	1611	9.07	<0.001
	ROI9: left supra marginal	−61.5	−52	26.5	673	9.01	<0.001
	ROI110: Temporal_Mid (dlpfc)	50.5	−34.5	2	363	7.92	<0.001
	ROI11: Frontal_Mid_2 (R)	40	25	40.5	83	4.77	0.029

*Note*: Coordinates represent the center of each region-of-interest sphere extracted for analyses along with corresponding cluster size (K), Ze (peak-level Z-equivalent score), and FWE–corrected *p* values. Maps were generated by xjView with initial *p* < 0.001 and cluster size = 40. ACC, anterior cingulate cortex; BA, Brodmann area; dlPFC, dorsolateral prefrontal cortex; FWE, family-wise error; KE, cluster size (number of voxels); L, left; MNI, Montreal Neurological Institute; R, right; vmPFC: ventromedial prefrontal cortex.

**Figure 3.**
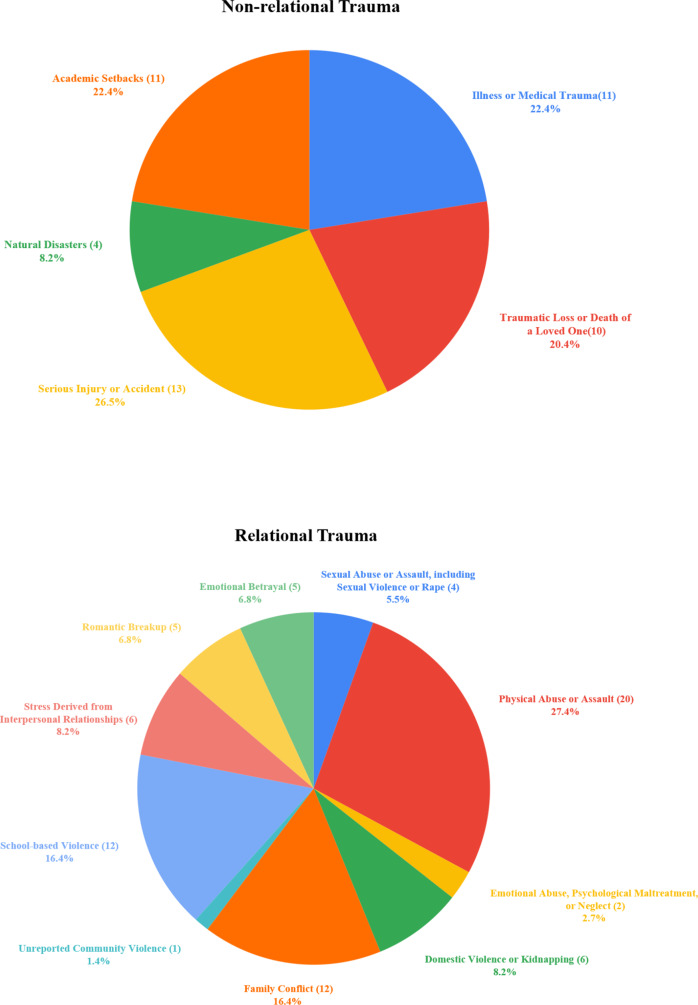
Nonrelational trauma (*n* = 57). The number of individuals endorsing each atypical traumatic event type is given in parentheses. Relational trauma (*n* = 79). The number of individuals endorsing each atypical traumatic event type is given in parentheses.

### Types and groups of trauma (ROI results)

A 2 × 2 between-subjects ANOVA was conducted for each ROI to examine the main effects of group (PTSD versus trauma exposure) and trauma type (relational versus non-relational), as well as their interaction. Type III sum of squares was applied, and homogeneity of variance was confirmed for all ROIs using Levene’s test (*p* > .20 for all analyses). Partial eta-squared (ηp^2^) was reported as the effect size, with values interpreted as follows: ηp^2^ ≥ 0.01 (small), ≥0.06 (medium), and ≥0.14 (large). (a) Implicit emotion regulation: in ROI3 (Right superior frontal gyrus [SFG]; Frontal_Sup_2), PTSD participants showed reduced activation compared to the trauma exposure group (*F* (1,117) = 3.971, *p* = 0.049, ηp^2^ = 0.033), with PTSD participants having a mean of (*M* = −0.41, *SD* = 0.94) and trauma exposure participants having a mean of (*M* = −0.76, *SD* = 1.09). (b) Emotion modulation by attention shifting: significant differences in brain activation were observed between patients with PTSD and the trauma exposure group, as well as across trauma types. ROI4 (left fusiform gyrus [LFG], BA37; fusiform) showed a significant main effect of group (*F* (1,117) = 9.71, *p* = 0.002, ηp^2^ = 0.077), with PTSD participants exhibiting lower activation (*M* = 1.67, *SD* = 1.68) compared to trauma-exposed controls (*M* = 2.69, *SD* = 1.85). A main effect of trauma type was also significant (*F* (1,117) = 4.49, *p* = 0.036, ηp^2^ = 0.037), with nonrelational trauma associated with higher activation (*M* = 2.40, *SD* = 1.97) than relational trauma (*M* = 1.76, *SD* = 1.63). A marginally significant interaction effect was observed in ROI4 (*F* (1,117) = 3.126, *p* = 0.080, ηp^2^ = 0.026). Similarly, in ROI6 (left SFG), PTSD participants exhibited lower activation than the trauma exposure group (*F* (1,117) = 4.059, *p* = 0.046, ηp^2^ = 0.034), with PTSD participants having a mean of (*M* = -0.59, *SD* = 1.13) compared to (*M* = −1.01, *SD* = 1.10) in the trauma exposure group. (c) Emotion modulation by appraisal: ROI8 (SFG) demonstrated a significant main effect of the diagnostic group (*F* (1,117) = 7.37, *p* = 0.008, ηp^2^ = 0.059), with PTSD participants exhibiting markedly reduced neural activation (*M* = 0.75, *SD* = 1.07) compared to trauma-exposed controls (*M* = 1.31, *SD* = 1.26). The effect size (ηp^2^ = 0.059) indicates a moderate association between diagnostic status and activation in this region, accounting for approximately 5.9% of the variance. All ROI results can be found in Supplementary Material A3.

### Correlations between ROI and clinical symptoms

The study revealed four key findings: (1) symptom severity (PCL-5, GAD-7, PHQ-9) showed significant negative correlations with social support (*r* = −0.353 to −0.417, *p* < 0.01). (2) ROI7 (LFG, BA37) exhibited negative associations with PTSD, anxiety, and depression severity (*r* = −0.244 to −0.311, *p* < 0.01) and strong anticorrelation with ROI1 (LFG; *r* = −0.538, *p* < 0.001). (3) ROI3 (right SFG, BA9) demonstrated positive correlations with anxiety and depression (*r* = 0.185–0.219, *p* < 0.05) and functional synergy with ROI2 (right inferior frontal gyrus, triangularis; *r* = 0.417, *p* < 0.001). All correlations of ROI results can be found in Supplementary Material A4.

## Dicussion

### General discussion of results

Aligned with the NHMS framework, this study provides novel evidence that PTSD-R and PTSD-NR differentially disrupt hierarchical self-processing during implicit emotion regulation. PTSD-R showed hypoactivation in the right SFG (ROI3), impairing self-schema integration, while PTSD-NR exhibited heightened survival circuit activity (e.g. fusiform gyrus, ROI4), amplifying threat monitoring. The absence of trauma-type-by-group interactions across most ROIs suggests that PTSD involves generalized predictive coding dysregulation – relational trauma distorts top-down priors (‘I am unsafe’), whereas nonrelational trauma heightens bottom-up prediction errors (‘unpredictable threats’). These findings extend explicit regulation studies by identifying trauma-specific neural biomarkers: relational PTSD disrupts DMN connectivity (SFG hypoactivation), while nonrelational PTSD drives limbic hypervigilance (ROI4 hyperactivation). This parallels NHMS-derived fMRI findings of mental-tier DMN dysfunction (e.g. mPFC hypoactivation) in relational PTSD (Scalabrini et al., 2024). Clinically, this dissociation supports targeted interventions – cognitive restructuring for DMN-based distortions in relational PTSD and interoceptive biofeedback for nonrelational hyperarousal. Despite cross-sectional limitations, these insights refine NHMS by linking trauma typology to hierarchical circuit dysfunction, informing precision psychiatry in PTSD. PTSD diagnosis, regardless of trauma type, is linked to severe psychopathology, with elevated PCL-5, GAD-7, and PHQ-9 scores (all *p* < 0.001, ηp^2^ > 0.50), supporting NHMS predictions of broad disruptions in survival and self-regulatory networks. Notably, while neuroimaging reveals trauma-specific alterations (e.g. fusiform hyperreactivity in nonrelational trauma), core PTSD symptoms converge into a common phenotype. Social support findings (*p* = 0.018) show PTSD-R survivors report lower support than PTSD-NR, aligning with DMN dysfunction and impaired self-other schemas. This may reflect mPFC hypoactivation – a DMN region supporting both self-referential and social-affiliative processing (Mars et al., 2012) – suggesting that PTSD-R involves disrupted integration of identity and interpersonal trust, consistent with the NHMS model. Reduced perceived social support in PTSD-R may thus mirror this neural dysfunction. However, trauma-exposed controls with relational trauma had higher support (*p* = 0.026), suggesting a potential ‘steeling effect’ in the absence of PTSD. These findings reinforce the NHMS framework: while trauma type differentially affects neural circuits, PTSD universally exacerbates psychopathology. Clinically, relational PTSD may benefit from social network rebuilding and cognitive restructuring, whereas nonrelational PTSD could respond better to sensory modulation. This study also identifies a tripartite prefrontal dysfunction hierarchy in emotion regulation and cognition – right dlPFC impairment disrupts executive control, left mPFC (ROI6) hinders self-referential integration, and ROI8 impairs emotional valence appraisal. These circuit-specific disruptions highlight neuromodulation targets, with repetitive transcranial magnetic stimulation (rTMS) over the right dlPFC (Masina, Tarantino, Vallesi, & Mapelli, 2019) potentially reducing comorbid anxiety and real-time fMRI neurofeedback targeting the left mPFC (Zotev & Bodurka, 2020) aiding self-referential coherence. Future research should integrate behavioral and neural markers to refine PTSD subtyping and treatment personalization.

### Fusiform gyrus and its interactive significance

The fusiform gyrus (LFG, BA37), a hub for high-level visual processing and threat detection (Weiner & Zilles, 2016), emerged as a critical locus differentiating both PTSD diagnostic status and trauma typology during attention shifting. In implicit emotion tasks, the brain regions involved in face processing differ from those associated with the facial perception model. Unlike explicit tasks, which engage regions responsible for recognizing facial identity and emotion, implicit tasks that focus on judging gaze direction activate only the fusiform gyrus and the inferior occipital gyrus (Colibazzi et al., 2010). Moreover, reduced fusiform gyrus (BA37) engagement may reflect impaired emotional face processing in trauma-related psychopathology, with its anticorrelation to default-mode regions suggesting dysregulated perceptual-self-referential balance.

A significant group effect (*F* = 9.71, *p* = 0.002, ηp^2^ = 0.077) showed PTSD-related fusiform hypoactivation, supporting its role in impaired threat appraisal (Killgore et al., 2014). Notably, a trauma-type effect (*F* = 4.49, *p* = 0.036) indicated heightened fusiform activation in nonrelational trauma survivors (*M* = 2.40 versus 1.76), suggesting enhanced sensory vigilance for environmental threats, whereas relational trauma survivors exhibited suppression, potentially reflecting avoidance of socially salient cues (Li, Li, Hu, Yang, & Luo, 2024; Liberzon et al., 2015). A marginal interaction (*F* = 3.13, *p* = 0.080) suggests that fusiform reactivity varies by trauma type and PTSD status. Non-relational trauma-exposed controls showed the highest activation, while relational patients with PTSD exhibited the lowest. This aligns with the neurocognitive specialization hypothesis: repeated interpersonal threats may downregulate fusiform responses to prevent hyperarousal, whereas nonrelational trauma reinforces hypervigilant visual scanning (Morey et al., 2015). This aligns with the NHMS framework’s extero-proprioceptive tier, where BA37 integrates visual inputs with somatic states to guide adaptive attention shifts (Scalabrini et al., 2024). Fusiform hypoactivation in PTSD – regardless of trauma type – may indicate impaired attention shifting from internal distress to external context, reinforcing maladaptive threat cycles (Liuzzi et al., 2024; Russman Block et al., 2020). This highlights the fusiform’s dual role as both a trauma-specific sensory adaptation marker and a transdiagnostic indicator of attentional dysregulation. Future research should explore fusiform-targeted interventions (e.g. gaze-contingent biofeedback) to address these anomalies.

### Other significant brain regions of neural circuits

#### Right dlPFC (Frontal_Sup_2, ROI3)

The right dlPFC, a hub for cognitive control and working memory (Friedman & Robbins, 2022; Menon & D’Esposito, 2022; Tang et al., 2019), exhibited reduced activation in PTSD participants during implicit emotion processing (*F* = 3.97, *p* = 0.049, ηp^2^ = 0.033). This hypoactivation aligns with its role in top-down regulation of limbic reactivity (Li et al., 2017; Soutschek & Tobler, 2020), suggesting a failure to recruit cognitive resources for automatic emotion suppression – a deficit transcending trauma type. The dlPFC’s engagement in implicit regulation is thought to reflect ‘covert’ cognitive effort (Fitzgerald, Kinney, Phan, & Klumpp, 2020; Webler et al., 2022), and its blunted activity in PTSD may perpetuate amygdala-driven hyperarousal. Notably, while trauma-exposed controls showed normative dlPFC engagement (*M* = −0.76), PTSD patients’ hypoactivation (*M* = −0.41) mirrors findings in generalized anxiety (Balderston et al., 2017; White, Makhoul, Teferi, Sheline, & Balderston, 2023), implying shared prefrontal dysregulation across stress-related disorders. This supports the NHMS framework’s assertion that mental-tier deficits (e.g. dlPFC-mediated executive dysfunction) represent a transdiagnostic PTSD vulnerability, independent of trauma etiology.

#### Left mPFC (Frontal_Sup_Medial, ROI6)

In the left mPFC – a key node of the DMN involved in self-referential processing (Lin, Callahan, & Moser, 2018; Molnar-Szakacs & Uddin, 2013) – PTSD participants demonstrated hypoactivation during attention shifting (*F* = 4.06, *p* = 0.046, ηp^2^ = 0.034). This region’s reduced engagement suggests impaired integration of self-relevant stimuli during attentional reorientation, potentially exacerbating rumination in PTSD (Paulus & Stein, 2010; Wong, Chen, Lee, Suen, & Hui, 2023). The absence of trauma-type effects here contrasts sharply with fusiform gyrus findings, highlighting a critical NHMS distinction: while sensory regions (e.g. fusiform) encode trauma-specific threat signatures, DMN hubs like the mPFC manifest *generalized* self-processing deficits. This aligns with Sripada et al. (2012) that PTSD disrupts the DMN’s baseline role in contextualizing threat within autobiographical frameworks.

#### Left mPFC (Frontal_Sup_Medial, ROI8)

The same mPFC subregion (ROI8) showed pronounced hypoactivation during emotion appraisal (*F* = 7.37, *p* = 0.008, ηp^2^ = 0.059), underscoring its dual role in both self-referential and evaluative processes. As a convergence zone for valence assessment (Chavez & Heatherton, 2015; Coley, Padilla-Coreano, Patel, & Tye, 2021), its dysfunction may explain PTSD patients’ to adaptively reappraise trauma-related stimuli – a deficit quantified by the moderate effect size (ηp^2^ = 0.059). Crucially, the mPFC’s engagement across both tasks reflects its integrative role in the NHMS – linking higher-order self-representation with affective evaluation from lower tiers. This dual function supports its position at the interface of introspective and sensory-affective processing (Chavez & Heatherton, 2015). This finding extends the work by Etkin, Egner, and Kalisch (2011)), who identified mPFC-amygdala decoupling in PTSD, but our results specify that mPFC deficits persist even in implicit contexts, independent of explicit cognitive effort.

### Research limitations

Several limitations must be considered. First, the relatively small sample size and the high proportion of local university students (72%) may lead to both limited statistical power and contextual homogeneity in trauma types, reducing generalizability. Second, trauma chronicity varies, complicating distinctions between acute and chronic adaptations. Future studies should specify posttrauma intervals or adopt longitudinal designs to clarify temporal dynamics. Third, reliance on a single fMRI task constrains functional interpretations; multimodal imaging and diverse paradigms could better isolate trauma-specific neural signatures. Finally, the cross-sectional design precludes causal inferences, highlighting the need for multisite longitudinal studies with harmonized protocols and experimental interventions.

## Conclusion

This study demonstrates that relational and nonrelational PTSD differentially affect hierarchical self-processing. Relational trauma disrupts metacognitive networks (SFG), impairing self-other distinction, while nonrelational trauma dysregulates survival circuits (FG), intensifying threat monitoring. The inverse dorsolateral prefrontal-default mode connectivity suggests predictive coding disruptions as a transdiagnostic mechanism. Reduced social support in relational PTSD highlights environmental influences on neurocognitive vulnerability. These findings support trauma-specific interventions: cognitive therapies for self-schema integration in relational PTSD and biofeedback for interoceptive regulation in nonrelational cases.

## Supporting information

Guo et al. supplementary material Guo et al. supplementary material

## Supplementary material

The supplementary material for this article can be found at http://doi.org/10.1017/S0033291725101505.
