# Supplementary material for: Neural mechanisms underlying implicit emotion regulation deficit in relational and nonrelational trauma PTSD: Insights from the Nested Hierarchical Model of Self
Source: Psychol Med. 2025 Aug 27;55:e248. doi: 10.1017/S0033291725101505 (PMC12404329; doi:10.1017/S0033291725101505)
Supplement: Guo et al. supplementary material [file S0033291725101505sup001.docx]

**Supplemental Material A1: Operational Criteria for Trauma Type Classification**

**Supplemental Material A2:** **Detailed fMRI task**

**Supplemental** **Material A3:** **Types and groups of trauma (ROI results)**

**Supplemental Material A4: Correlations between ROI and clinical symptoms**

**Supplemental Material A1: Operational Criteria for Trauma Type Classification**

**1. Theoretical Foundations**
Trauma typology was classified as **interpersonal** (relational harm) or **non-interpersonal** (externally caused) based on DSM-5 Criterion A definitions, prior research frameworks (Alisic et al., 2014; Kerig et al., 2009; Hughesdon et al., 2021), and participants’ qualitative self-reports of their index traumatic events. Interpersonal trauma aligns with the WHO’s violence taxonomy (Krug et al., 2002), emphasizing intentional harm by individuals or small groups, whereas non-interpersonal trauma reflects environmental or accidental events.

**2. Data Sources and Event Screening**

- **Standardized Instruments**:
  - *LEC-5*: Participants reported exposure to 17 DSM-5 Criterion A events (Weathers et al., 2013). Item 17 was modified to capture pandemic-related stressors (e.g., COVID-19 outbreaks), contextualizing Wuhan’s epidemiological crisis.
  - *LECQT*: Supplementary open-ended questions identified non-Criterion A events causing persistent distress (e.g., "Have you experienced events leading to intrusive memories or avoidance behaviors?").

**3. Event Prioritization Protocol**
Events were hierarchically prioritized using:

1. **Direct Experience** (Criterion A1: e.g., physical assault)
2. **Witnessed Events** (Criterion A2: e.g., observing violence)
3. **Vicarious Exposure** (Criterion A3: e.g., learning of a loved one’s trauma)
   For participants reporting multiple traumas:

- *Predominance Rule*: Classification followed majority type (>50% events per category).
- *Tiebreaker*: Equal distributions were flagged (code 2) and resolved via consensus review.

**4. Operational Definitions and Examples**

- **Interpersonal Trauma** (Code 1): Intentional human perpetration
  - *Subtypes*: Sexual assault, domestic violence, emotional abuse, bullying, betrayal trauma (e.g., intimate partner deception), familial neglect.
  - *Exclusion*: Non-violent interpersonal conflicts (e.g., routine workplace disagreements).
- **Non-Interpersonal Trauma** (Code 0): External/environmental causes
  - *Subtypes*: Natural disasters, accidental injuries, medical trauma, non-violent bereavement, pandemic-related stressors.
  - *Exclusion*: Ordinary life stressors (e.g., academic pressure without traumatic impact).

**5. Coding Procedures and Reliability**
Three PTSD-specialized clinicians independently coded events using:

1. **Blinded Initial Coding**: Achieved high inter-rater reliability (κ = 0.84, *p*<0.001).
2. **Consensus Conference**: Discrepancies (3.7% of cases) were resolved via structured discussion, referencing DSM-5 criteria and WHO guidelines.
3. **Final Adjudication**: Ambiguous cases (<1.5%) underwent third-tier review with a senior trauma psychologist.

**6. Analytical Workflow**
Final classifications were analyzed in SPSS 24 (IBM Corp) using:

- Chi-square tests for group comparisons.
- Logistic regression to assess trauma type associations with clinical outcomes.

**7. Validation Against Literature**
This protocol integrates:

- Kerig et al.’s (2009) emphasis on relational rupture in interpersonal trauma.
- Vibhakar et al.’s (2019) distinction between human-perpetrated vs. environmental trauma.
- Hughesdon et al.’s (2021) framework linking interpersonal trauma to psychosocial dysfunction.

**8. Exclusion Criteria**
Non-traumatic events were systematically excluded through:

1. **Triage Screening**: Cross-referencing LEC-5/LECQT responses with DSM-5 Criterion A.
2. **Coder Training**: Clinicians completed a 10-hour workshop on distinguishing traumatic vs. stressful events.

**9.** **PCL-5 score**

- PCL-5 > 31 → *referred for diagnostic interview (not automatic PTSD diagnosis)*
- PCL-5 < 31 → *assigned to trauma-exposed group if a Criterion A trauma was reported*
- PTSD diagnosis → *based on structured clinical interview conducted by trained personnel under psychiatrist supervision*

**Supplemental Material A2:** **Detailed fMRI task**

The Shifted Attention Emotion Appraisal Task (SEAT) was used to investigate neural functions associated with naturalistic emotion regulation processes, including implicit emotional processing, attention modulation of emotion, and emotion modulation by appraisal. Participants viewed compound images of neutral and threatening (fearful and angry) faces superimposed on indoor and outdoor scenes. Before each image, one of three cues appeared: “Male/Female” (identify the gender of the face), to probe implicit emotional processing; “Indoor/Outdoor” (is the scene indoor or outdoor), to probe emotion modulation by attention shifting; or “Like/Dislike” (do you like or dislike the face), to probe emotion modulation by appraisal. Neutral faces alone and places alone trials were also presented throughout the task to control for brain activation associated with simply viewing faces and scenes.

Facial images from the Chinese emotional picture library were selected as the experimental materials of this study. The picture stimulus in the task is presented using E-Prime. The image is a composite face/scene image made up of 20 angry, 20 fearful, and 20 neutral expressions superimposed on 20 architectural scenes (10 indoor, 10 outdoor). To locate the brain regions involved in face and place processing, another 10 neutral faces and 10 indoor or outdoor scenes were used as non-composite images. A total of 80 images are required. Participants viewed the composite images and answered three different questions about each image: 1) pay attention to the face in the composite image and determine whether it was male or female (male/female condition); 2) Pay attention to the scene on the composite picture and determine whether it is indoor or outdoor (indoor/outdoor conditions); 3) Pay attention to the face on the composite image and judge whether you like or dislike the face (like/dislike condition). To study the implicit processing of emotions, attention to emotional stimuli was maintained in the male/female condition without participating in the evaluation. The other two conditions involved both implicit processing of emotion and two kinds of unintentional emotion regulation: 1) attention redirection (indoor/outdoor conditions); and 2) Evaluation (like/dislike conditions). Each composite diagram is presented three times, once for each condition, and the condition type is presented randomly (180 trials in total). In both male/female and indoor/outdoor conditions, the correct response involves accurately identifying the gender of a face (male/female) or the location of a scene (indoor/outdoor). The presentation of a non-composite image (showing only a face or scene) was a total of 40 trials, and the subjects had to decide whether the image was a face or a place. A total of 220 trials were presented randomly in 4 blocks (55 trials each). The experiment consisted of 3-8 seconds of central fixation point "+", 750ms judgment prompt and 250ms blank screen, and finally 1500ms composite picture. Flowchart of the SEAT experimental paradigm See Figure 1.


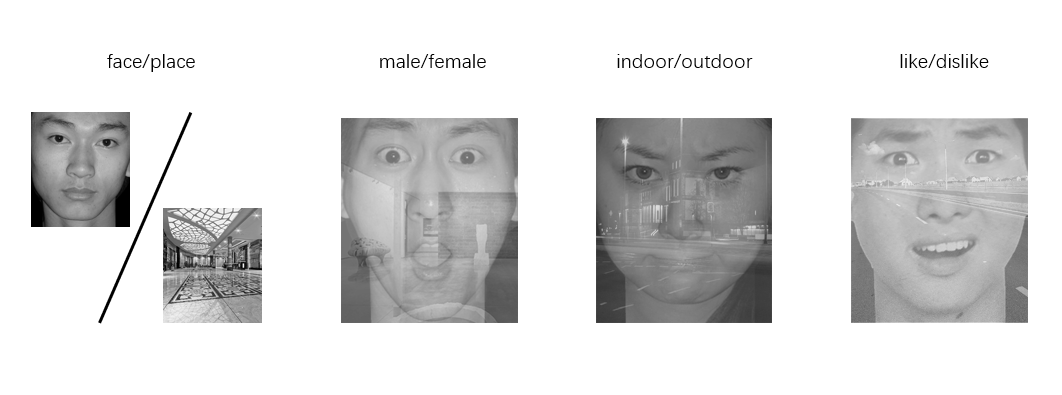

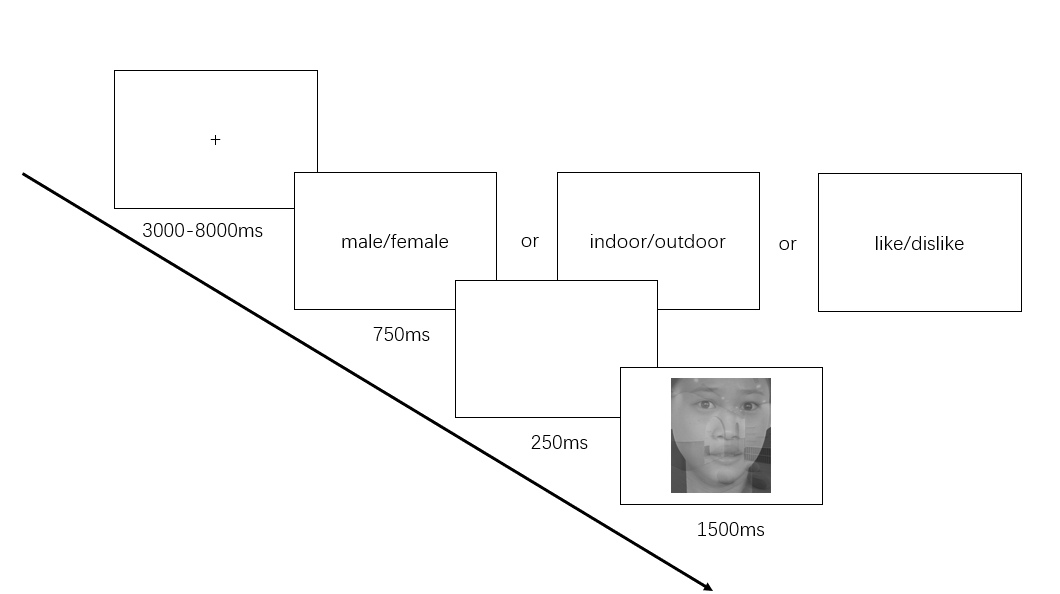


Figure1. Flowchart of the SEAT experimental paradigm

**Supplemental Material A3:** **Types and groups of trauma (ROI results)**

| **ROI** | **Group Effect** | **Trauma Type Effect** | **Interaction Effect** | | **PTSD_M (SD)** | **HC_M (SD)** |
| --- | --- | --- | --- | --- | --- | --- |
| 1 | *F*(1,117)=1.31, *p*=.255 | *F*(1,117)=0.13, *p*=.724 | *F*(1,117)=1.01, *p*=.317 | | -1.09 (1.54) | -1.38 (1.26) |
|  | ηp²=.011 | ηp²=.001 | ηp²=.009 | |  |  |
| 2 | *F*(1,117)=2.24, *p*=.137 | *F*(1,117)=0.00, *p*=.970 | *F*(1,117)=0.25, *p*=.621 | | 0.45 (1.11) | 0.80 (1.20) |
|  | ηp²=.019 | ηp²=.000 | ηp²=.002 | |  |  |
| **3** | ***F*(1,117)=3.97, *p*=.049** | *F*(1,117)=0.63, *p*=.430 | *F*(1,117)=3.43, *p*=.066 | | -0.41 (0.94) | -0.76 (1.09) |
|  | **ηp²=.033** | ηp²=.005 | ηp²=.029 | |  |  |
| **4** | ***F*(1,117)=9.71, *p*=.002** | ***F*(1,117)=4.49, *p*=.036** | *F*(1,117)=3.13, *p*=.080 | | 1.67 (1.68) | 2.69 (1.85) |
|  | **ηp²=.077** | **ηp²=.037** | ηp²=.026 | |  |  |
| 5 | *F*(1,117)=3.79, *p*=.054 | *F*(1,117)=1.32, *p*=.253 | *F*(1,117)=1.64, *p*=.203 | | 0.41 (0.86) | 0.74 (0.99) |
|  | ηp²=.031 | ηp²=.011 | ηp²=.014 | |  |  |
| **6** | ***F*(1,117)=4.06, *p*=.046** | *F*(1,117)=0.53, *p*=.467 | *F*(1,117)=1.29, *p*=.259 | | -0.59 (1.13) | -1.01 (1.10) |
|  | **ηp²=.034** | ηp²=.005 | ηp²=.011 | |  |  |
| 7 | *F*(1,117)=2.76, *p*=.099 | *F*(1,117)=0.26, *p*=.611 | *F*(1,117)=2.07, *p*=.153 | | 0.37 (0.91) | 0.63 (0.91) |
|  | ηp²=.023 | ηp²=.002 | ηp²=.017 | |  |  |
| **8** | ***F*(1,117)=7.37, *p*=.008** | *F*(1,117)=1.68, *p*=.198 | *F*(1,117)=3.05, *p*=.084 | | 0.75 (1.07) | 1.31 (1.26) |
|  | **ηp²=.059** | ηp²=.014 | ηp²=.025 | |  |  |
| 9 | *F*(1,117)=1.59, *p*=.196 | *F*(1,117)=1.06, *p*=.304 | *F*(1,117)=0.95, *p*=.332 | | 0.61 (0.99) | 0.94 (0.88) |
|  | ηp²=.013 | ηp²=.009 | ηp²=.008 | |  |  |
| 10 | *F*(1,117)=1.08, *p*=.285 | *F*(1,117)=0.35, *p*=.553 | *F*(1,117)=0.00, *p*=.999 | | 0.59 (0.94) | 0.92 (1.13) |
|  | ηp²=.009 | ηp²=.003 | ηp²=.000 | |  |  |
| 11 | *F*(1,117)=3.18, *p*=.077 | *F*(1,117)=0.01, *p*=.906 | *F*(1,117)=1.98, *p*=.163 | | 0.33 (1.05) | 0.66 (1.15) |
|  | ηp²=.026 | ηp²=.000 | ηp²=.017 | |  |  |
| **ηp² (Partial Eta-Squared)**: Interpreted as small (≥.01), medium (≥.06), large (≥.14) | | | |  |  |  |
| Significant effects (p < .05) bolded; trends (.05 ≤ p ≤ .10) italicized. | | | |  |  |  |
| M = Mean (z-scored activation), SD = Standard Deviation. | | | |  |  |  |

**Supplemental Material A4: Correlations between ROI and clinical symptoms**

| **Measure** | **Effect** | ***F*(df)** | ***p*** | **ηp²** | **Bootstrapped 95% CI** | **Descriptives (M ± SD)** |
| --- | --- | --- | --- | --- | --- | --- |
| **PCL-5** | Group | 509.10 (1,118) | <.001 | 0.812 | [32.79, 41.24] | PTSD: 42.94 ± 9.93; HC: 5.86 ± 4.20 |
|  | Trauma Type | 0.07 (1,118) | 0.799 | 0.001 | [-2.13, 2.85] |  |
|  | Group × Trauma | <0.001 (1,118) | 0.988 | 0 | [-4.92, 5.30] |  |
| **GAD-7** | Group | 138.80 (1,118) | <.001 | 0.541 | [7.37, 11.32] | PTSD: 10.95 ± 4.83; HC: 1.74 ± 1.45 |
|  | Trauma Type | 0.23 (1,118) | 0.636 | 0.002 | [-0.30, 1.43] |  |
|  | Group × Trauma | 0.05 (1,118) | 0.819 | 0 | [-2.76, 2.19] |  |
| **PHQ-9** | Group | 173.81 (1,118) | <.001 | 0.596 | [8.72, 13.15] | PTSD: 13.54 ± 5.22; HC: 2.29 ± 1.70 |
|  | Trauma Type | 0.55 (1,118) | 0.46 | 0.005 | [-0.79, 1.45] |  |
|  | Group × Trauma | 0.10 (1,118) | 0.749 | 0.001 | [-2.23, 3.15] |  |
| **Social Support** | Group | 16.74 (1,118) | <.001 | 0.124 | [-15.06, 4.89] | PTSD: 49.71 ± 14.01; HC: 63.10 ± 19.75 |
|  | Trauma Type | 1.41 (1,118) | 0.238 | 0.012 | [-0.85, 23.02] |  |
|  | Group × Trauma | 5.78 (1,118) | 0.018 | 0.047 | [-28.05, -1.48] | Relational: PTSD 48.35 ± 14.17 vs. HC 68.36 ± 18.20 |

**References**

Alisic, E., Zalta, A. K., Van Wesel, F., Larsen, S. E., Hafstad, G. S., Hassanpour, K., & Smid, G. E. (2014). Rates of post-traumatic stress disorder in trauma-exposed children and adolescents: Meta-analysis. *British Journal of Psychiatry*, *204*(5), 335–340. https://doi.org/10.1192/bjp.bp.113.131227

Kerig, P. K., Ward, R. M., Vanderzee, K. L., & Arnzen Moeddel, M. (2009). Posttraumatic Stress as a Mediator of the Relationship Between Trauma and Mental Health Problems Among Juvenile Delinquents. *Journal of Youth and Adolescence*, *38*(9), 1214–1225. https://doi.org/10.1007/s10964-008-9332-5

Krug, E. G., Mercy, J. A., Dahlberg, L. L., & Zwi, A. B. (2002). The world report on violence and health. The lancet, 360(9339), 1083-1088.

Hughesdon, K. A., Ford, J. D., Briggs, E. C., Seng, J. S., Miller, A. L., & Stoddard, S. A. (2021). Interpersonal Trauma Exposure and Interpersonal Problems in Adolescent Posttraumatic Stress Disorder. *Journal of Traumatic Stress*, *34*(4), 733–743. https://doi.org/10.1002/jts.22687
